# Incidental Finding of a Lipoleiomyoma During Robotic Inguinal Hernia Repair: A Case Report

**DOI:** 10.7759/cureus.40635

**Published:** 2023-06-19

**Authors:** Shohab K Virk, Edilin Lopez, Katrina M Pardo, Anthony M Gonzalez

**Affiliations:** 1 Department of Surgery, Baptist Hospital of Miami, Miami, USA; 2 Department of Surgery, Baptist Health South Florida, Miami, USA

**Keywords:** robotic surgery, incidental findings, fibroids, inguinal hernia, lipoleiomyoma, uterus

## Abstract

Lipoleiomyomas are rare, fatty variants of leiomyomas (commonly referred to as fibroid), which are frequently found in the uterine corpus and cervix. Here, we present a case of a robotic inguinal hernia repair with resection of an incidental lipoleiomyoma.

A 74-year-old woman presented to the office with complaints of pain and a palpable mass in the right inguinal region. Physical examination revealed tender, moderate-to-large bilateral inguinal hernias. Robotic bilateral inguinal hernia repair with mesh was performed. Intraoperatively, a mass measuring 4 × 3 cm was noted near the round ligament of the uterus. The mass was encapsulated without invading any surrounding structures. The mass was resected and sent to the histopathology department. The pathological evaluation identified a leiomyoma filled with mature adipocytes, compatible with the diagnosis of an extrauterine lipoleiomyoma.

Lipoleiomyoma incidentally found in the inguinal canal is extremely rare. The medical literature regarding this incidental finding is limited. Resection of the mass was easily performed using the same robotic instruments as used for the inguinal hernia repair.

## Introduction

Although uterine leiomyomas or fibroids can be found in 20-30% of women over 35 years of age [1], uterine lipoleiomyomas are atypical, having an incidence of about 0.03-0.2% [2]. Lipoleiomyomas are a rare benign variant of leiomyomas that contain mature adipocytes. They are most commonly located in the uterus but can also arise from the smooth muscle of the gastrointestinal tract. The pathogenesis is multifactorial, ranging from metastasis from other sites such as the retroperitoneum to iatrogenic during surgeries. According to a literature review on PubMed and Google Scholar, Byun et al. previously reported a case of an ectopic lipoleiomyoma incidentally found during a laparoscopic inguinal hernia repair. To our knowledge, this is the second case ever reported [3]. It is important to remove lipoleiomyomas upon incidental findings due to the potential for malignant transformation [4]. The lipoleiomyoma variant of leiomyoma may be associated with certain risk factors related to metabolic disorders, such as diabetes mellitus [4]. Most lipoleiomyomas remain benign, causing space-occupying lesions which lead to pain and discomfort; however, there is a rare chance that they can undergo malignant transformation [4]. This is the main reason for the removal of this incidental finding.

## Case presentation

A 74-year-old woman was referred to the surgical outpatient office for the evaluation of recurrent inguinal hernias. The general and systematic physical examinations revealed tense, moderate-to-large bilateral inguinal hernias on Valsalva approximately 3 cm in size which reduced on palpation. The diagnosis was purely clinical and no investigations were ordered. The patient had a previous surgical history of a hysterectomy, open appendectomy, abdominoplasty, and initial inguinal hernia repairs six years prior.

**Figure 1:**
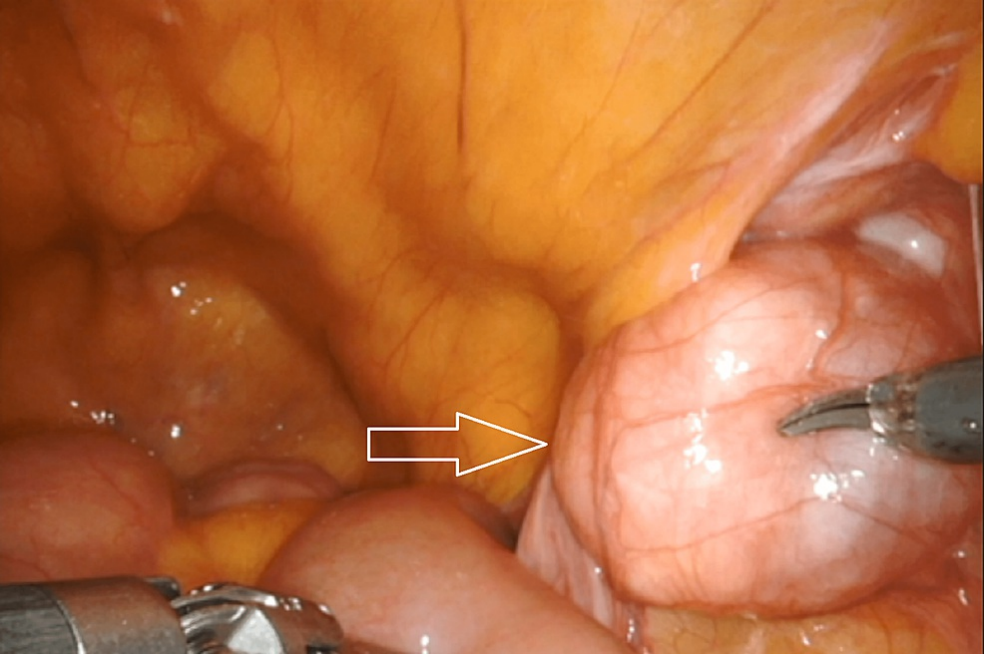
Intraoperative findings showing a mass attached to the right round ligament.

**Figure 2:**
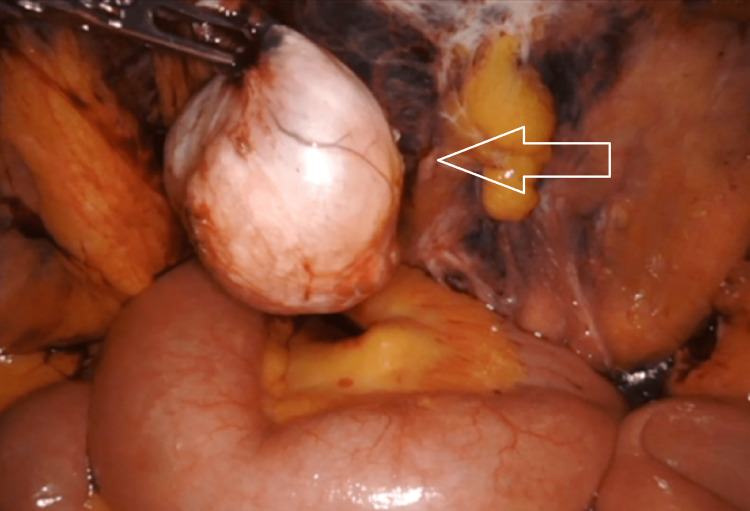
Intraoperative findings showing the excised mass.

**Figure 3:**
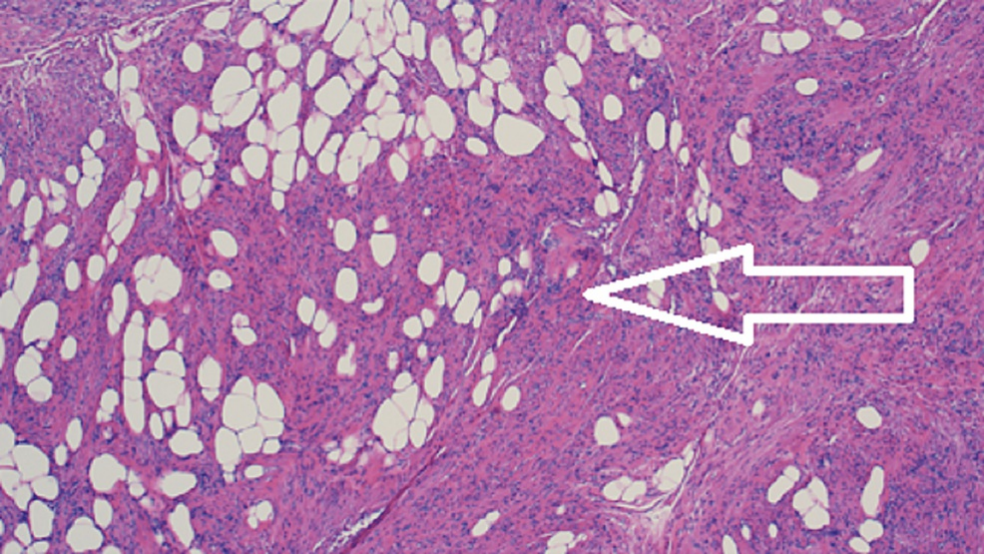
Microscopy using hematoxylin and eosin stain (10×) showing lipoleiomyomas made up of two components, smooth myocytes and mature adipocytes.

A decision was made to perform a robotic bilateral inguinal hernia repair with mesh. Intraoperatively, a mass measuring 4 × 3 × 1.8 cm was noted in the right inguinal canal of Nuck, as the round ligament of the uterus exited the internal ring. The mass was encapsulated and did not invade any surrounding structures (Figure 1). The mass was resected and sent for histopathological analysis (Figure 2). The histopathological evaluation identified a well-circumscribed leiomyoma filled with a mixture of fascicles of spindled smooth muscles with mature adipocytes, compatible with the diagnosis of an extrauterine lipoleiomyoma (Figure 3). The immunohistochemistry showed that the tumor cells were strongly positive for desmin, smooth muscle antibody, and S-100 and were negative for CD34, epithelial membrane antigen, CD99, vimentin, human melanoma black-45, and beta-catenin. Ki67 proliferation index was virtually absent at less than 1%. The postoperative course was uneventful and the patient was discharged a few hours after surgery based on the modified Aldrete score. During the two-week follow-up in the office, the patient’s incisions were healing and there was no swelling or mass in the inguinal areas. She was instructed to follow up if any concerns arose in the surgical areas. Although the patient did not have a need to follow up in our surgical office, she has been followed by other specialists within our medical group for three years after surgery.

## Discussion

Lipoleiomyomas are rare, fatty variants of leiomyomas (or fibroids) and were first described by Meis in 1991 as a leiomyoma with an additional fatty component [2]. Inguinal hernias are more common in men than in women. The lifetime risk for a woman developing an inguinal hernia is around 1.9% [5]. Femoral hernias are four times more common in females, accounting for 16.7-37% of hernias found in females [6]. Lipoleiomyomas should be considered in the differential diagnosis, and a high degree of suspicion is required to be able to identify this pathology.

Lipoleiomyomas are known to be benign and can be treated conservatively [2]. The surgical options available as a treatment for uterine lipoleiomyomas include myomectomy, hysterectomy, myolysis, tumor embolization, and radiofrequency ablation [2]. Extrauterine tumors are excised with a rim of normal tissue [3-5]. To our knowledge, there is only one other report of an ectopic lipoleiomyoma in the inguinal region [7].

Lipoleiomyomas are discovered incidentally during other procedures or when they cause discomfort [8]. Unlike other masses, these do not invade the local tissues but rather have a space-occupying effect leading to symptoms [9]. Although a CT or MRI can be utilized to diagnose this pathology, in our case, it was diagnosed incidentally.

Inguinal swelling is typically attributed to inguinal hernias; however, other inguinal masses should also be in the differential diagnosis in women as recurrence and/or malignancies can arise from these rare tumors. There have been reports of other masses found incidentally during inguinal hernia repairs such as Meckel’s diverticulum and gastrointestinal stromal tumors [10,11].

## Conclusions

This is a case of a lipoleiomyoma incidentally found in the inguinal canal during a bilateral inguinal hernia repair. To our knowledge, this is one of the few rare cases reported in the medical literature. Resection of the mass was easily performed using the same robotic instruments as those used for the inguinal hernia repair. However, we recommend broadening the scope of differential diagnosis when it comes to cases of inguinal masses in women.
